# Clustering of Unhealthy Lifestyle and the Risk of Adverse Events in Patients With Atrial Fibrillation

**DOI:** 10.3389/fcvm.2022.885016

**Published:** 2022-07-04

**Authors:** So-Ryoung Lee, Eue-Keun Choi, Sang-Hyeon Park, Seung-Woo Lee, Kyung-Do Han, Seil Oh, Gregory Y. H. Lip

**Affiliations:** ^1^Department of Internal Medicine, Seoul National University Hospital, Seoul, South Korea; ^2^Department of Internal Medicine, Seoul National University College of Medicine, Seoul, South Korea; ^3^Department of Medical Statistics, College of Medicine, The Catholic University of Korea, Seoul, South Korea; ^4^Statistics and Actuarial Science, Soongsil University, Seoul, South Korea; ^5^Liverpool Center for Cardiovascular Science, University of Liverpool and Liverpool Chest and Heart Hospital, Liverpool, United Kingdom; ^6^Department of Clinical Medicine, Aalborg University, Aalborg, Denmark

**Keywords:** atrial fibrillation, lifestyle, stroke, myocardial infarction, heart failure

## Abstract

**Background:**

Little is known regarding the risk of clinical outcomes depending on the clustering of lifestyle behaviors after atrial fibrillation (AF) diagnosis. This study evaluated the association between a cluster of healthy lifestyle behaviors and the risk of adverse outcomes in patients with AF.

**Methods:**

Using the Korean National Insurance Service database, patients who were newly diagnosed with AF between 2009 and 2016 were included. A healthy lifestyle behavior score (HLS) was calculated by assigning 1 point each for non-current smoking, for non-drinking, and for performing regular exercise from the self-reported questionnaire in health examinations. The primary outcome was defined as major adverse cardiovascular event (MACE), including ischemic stroke, myocardial infarction, and hospitalization for heart failure.

**Results:**

A total of 208,662 patients were included; 7.1% in HLS 0, 22.7% in HLS 1, 58.6% in HLS 2, and 11.6% in HLS 3 groups. Patients with HLS 1, 2, and 3 were associated with a lower risk of MACE than those with HLS 0 (adjusted hazard ratio [95% confidence interval (CI)]: 0.788 [0.762–0.855], 0.654 [0.604–0.708], and 0.579 [0.527–0.636], respectively). After propensity score weighting, consistent results were observed. The risk reduction of healthy lifestyle combinations was consistently observed in various subgroups, regardless of the CHA_2_DS_2_-VASc score and oral anticoagulant use.

**Conclusion:**

Increased number of healthy lifestyle behaviors was significantly associated with lower MACE risk in patients with new-onset AF. These findings support the promotion of a healthy lifestyle to reduce the risk of adverse events in patients with AF.

## Introduction

Globally, the prevalence of atrial fibrillation (AF) is increasing with an aging population (1–3). AF is associated with an increased risk of stroke, heart failure, and death, and increases the overall healthcare burden (3–6). Therefore, the optimal management of AF, including AF burden reduction and stroke prevention, is crucial for improving outcomes and reducing the AF-related healthcare burden (7). Many studies have studied optimal oral anticoagulation treatment and better symptom care including rhythm and rate control in patients with AF; (8–10) however, lifestyle-related factors that play a role as modifiable risk factors in AF management are still generally underrecognized and understudied, especially in relation to clinical outcomes.

In previous studies, each component of unhealthy lifestyle behaviors such as smoking, alcohol consumption, and lack of regular exercise were individually associated with an increased risk of AF burden, thromboembolic events, and all-cause death (11–15). Although unhealthy or healthy lifestyle behaviors tend to be clustered, studies on the risk of clinical outcomes depending on how lifestyle behaviors are managed after AF diagnosis remain limited (16–19).

This study aimed to evaluate the clustering of healthy lifestyle behaviors in patients who were newly diagnosed with AF and the impact of such accumulation of multiple healthy lifestyle behaviors on the risk of AF-related adverse clinical outcomes.

## Materials and Methods

### Data Source and Study Population

The National Medical Claims Database, linked with the National Health Screening Examination Database established by the Korean National Health Insurance Service (NHIS) was used (20, 21). Briefly, the Korean NHIS provides universal, comprehensive, and mandatory medical coverage for the entire Korean population (approximately 50 million). The Korean NHIS database includes all the information about enrollees’ medical use, including demographic information, diagnoses, examinations, prescription records, procedures, and operations for inpatient and outpatient services. Diagnoses were coded based on the *International Classification of Diseases, Tenth Revision, Clinical Modification* codes. The Korean government provides a national health screening examination and recommends that all Korean adults receive examinations every 1 or 2 years. The health examination included anthropometric measurements, physical examinations, regular blood tests, and questionnaires on lifestyle behaviors and medical history.

**FIGURE 1:**
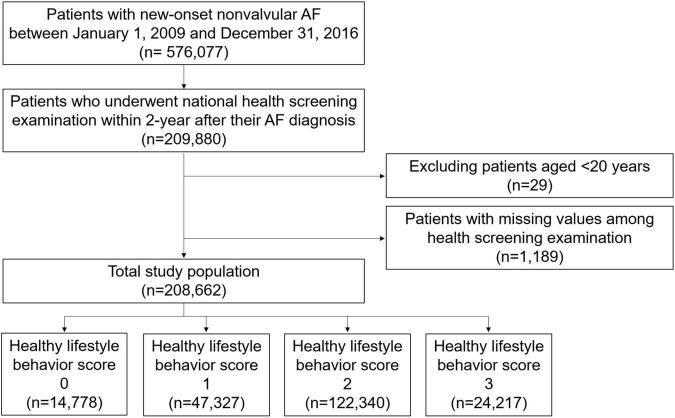
Study flow. AF, atrial fibrillation.

Among the patients who were newly diagnosed with non-valvular AF between 1 January 2009 and 31 December 2016 (*n* = 576,077), we included patients who underwent a national health screening examination within 2 years after their AF diagnosis (*n* = 209,880) (Figure 1). After excluding patients aged < 20 years and those with missing values among health screening examinations, 208,662 patients were finally included in this analysis.

This study was exempt from review by the Seoul National University Hospital Institutional Review Board (E-2103-006-1200). All data and materials have been made publicly available at the National Health Insurance Sharing Service and can be accessed at http://nhiss.nhis.or.kr/bd/ab/bdaba000eng.do.

### Definition of Healthy Lifestyle Behavior Score (HLS)

Lifestyle behaviors were assessed using self-reported questionnaires during health screening examinations. Each lifestyle was classified as follows: (1) smoking status as currently smoking or not; (2) alcohol consumption as a current drinker or non-drinker; and (3) regular exercise as performing moderate physical activity ≥ 5 times per week or vigorous physical activity ≥ 3 times per week and lack of regular exercise in the absence of regular exercise (19, 22). The frequency and intensity of physical activity were assessed using questionnaires. Moderate intensity of physical activity was defined as ≥ 30 min per day of brisk walking, dancing, or gardening, and vigorous intensity of physical activity was defined as ≥ 20 min per day of running fast, cycling, or aerobics (23).

To estimate the impact of each unhealthy lifestyle behavior on the risk of MACE, we evaluated the association between each unhealthy lifestyle behavior (with various dose or intensity) and the risk of MACE in the study population using multivariable Cox analysis (Supplementary Table 1). Based on the results of preliminary analyses, current smoker, current drinker, and non-regular exerciser were defined as patients with unhealthy lifestyle behaviors who were at significantly higher risk of MACE. Thus, in the present study, a healthy lifestyle behavior score (HLS) was calculated by assigning 1 point each for non-current smokers, non-drinkers, and performing regular exercise. The study population was categorized into four groups according to HLS from patients with HLS 0 who did not have any healthy lifestyle behaviors to patients with HLS 3 who met all three healthy lifestyle behaviors.

### Covariates

Demographic information including age and sex, comorbidities including hypertension, diabetes mellitus, dyslipidaemia, heart failure, prior ischemic stroke, prior myocardial infarction, peripheral artery disease, chronic obstructive pulmonary disease, cancer, and chronic kidney disease were assessed based on the diagnoses of medical claims and health screening examination database. The CHA_2_DS_2_-VASc score was calculated based on comorbidities (7). Detailed definitions of the comorbidities are presented in Supplementary Table 2 (2, 21, 24). Prescription records of claims database obtained medications including oral anticoagulants (warfarin or non-vitamin K antagonist oral anticoagulant), antiplatelet agents, and statins. Body mass index (BMI) was calculated as body weight in kilograms divided by the square of height in meters (kg/m^2^). Systolic and diastolic blood pressure, fasting glucose, and glomerular filtration rate were obtained from the health screening examination. Low income was defined as household income lower than 25%.

### Follow-Up and Study Outcomes

The primary outcome was the first occurrence of major adverse cardiovascular events (MACE), including ischemic stroke, myocardial infarction, and hospitalization for heart failure. Secondary outcomes included the individual components of MACE and all-cause deaths. Detailed definitions of the clinical outcomes are presented in Supplementary Table 2 (21, 24, 25). Patients were followed up starting from the index date (at the health screening examination date) until the occurrence of the study outcomes, death, or 31 December 2017 whichever came first.

### Statistical Analysis

Continuous variables were presented as mean and standard deviation and categorical variables were presented as number and percentage. Baseline characteristics across patient groups were compared using the Cochran-Armitage trend test for categorical variables and a linear trend test using a generalized linear model for continuous variables. The number of events was calculated as the incidence rate during the follow-up period divided by 100 person-years at risk. The risk of clinical outcomes with HLS was analyzed using the Cox proportional hazards regression model. The hazard ratio (HR) and 95% confidence intervals (CIs) for primary and secondary outcomes were analyzed using the HLS 0 group as the reference group. Model 1 was unadjusted; model 2 was adjusted for age and sex; and model 3 was further adjusted for hypertension, diabetes mellitus, dyslipidaemia, heart failure, prior ischemic stroke, prior myocardial infarction, peripheral artery disease, chronic obstructive pulmonary disease, cancer, chronic kidney disease, CHA_2_DS_2_-VASc score, use of oral anticoagulants, antiplatelet agents, and statins, BMI, and low income. The proportional hazards assumption was graphically evaluated with a log minus log graph and confirmed with Schoenfeld residuals for Cox models. Parallel log minus log survival curves and random patterns in Schoenfeld residuals were found, indicating no major deviation from the proportionality assumption, and the test was not statistically significant. The variance inflation factor (VIF) was used to assess multi-collinearity. Between the covariates, there was no significant collinearity (VIF = 1.005–2.492).

To provide complementary analyses for balancing among patient groups with different HLS, we performed inverse probability of treatment weighting (IPTW) using stabilized weights calculated from the propensity scores as a sensitivity analysis (26). The covariates included in model 3 were used for the propensity score calculation. We evaluated the maximum absolute standardized difference (ASD) of covariates to confirm the balance of the different groups. A maximum ASD of > 0.1 (10%) indicates an imbalance in a covariate (27, 28). The weighted incidence rate (per 100 person-years) and weighted cumulative incidence curves using the Kaplan–Meier method and log-rank test for primary and secondary outcomes were evaluated. The risk for primary and secondary outcomes of the different HLS groups was evaluated using weighted Cox proportional hazards models with IPTW. When the maximum ASD was > 0.1, the covariate was included in the Cox proportional hazard model for further adjustment.

For the primary outcome, subgroup analyses were performed for age (< 65, 65 to < 75, and ≥ 75 years), sex, CHA_2_DS_2_-VASc score (< 3 and ≥ 3), presence of prior history of ischemic stroke, and use of oral anticoagulants.

All analyses were two-tailed, and statistical significance was defined as *P* < 0.05. Statistical analyses were conducted using SAS (version 9.4; ASA Institute Inc., Cary, NC, United States).

## Results

### Baseline Characteristics

A total of 208,662 patients with AF who were available for national health screening exam data were included in this analysis (Figure 1). The mean duration between AF diagnosis and the national health screening exam was 333 ± 203 days. The proportions of patients with 0, 1, 2, and 3 HLS were 7.1%, 22.7%, 58.6%, and 11.6%, respectively, and the baseline characteristics according to HLS are presented in Table 1. The baseline characteristics of the study population are summarized in Supplementary Table 3.

**TABLE 1:** Baseline characteristics of the study population.

	Before IPTW					After IPTW				
	Healthy lifestyle behavior score				Maximum ASD	Healthy lifestyle behavior score				Maximum ASD
	0 (*n* = 14,778)	1 (*n* = 47,327)	2 (*n* = 122,340)	3 (*n* = 24,217)		0 (*n* = 14,363)	1 (*n* = 45,841)	2 (*n* = 123,866)	3 (*n* = 23,905)	
**Age, years**										
Mean ± SD	54.3 ± 12.4	59.6 ± 12.8	66.3 ± 12.1	64.6 ± 10.8	0.97	63.2 ± 12.6	63.0 ± 12.7	63.3 ± 13.4	63.8 ± 11.7	0.06
< 65	79.6	63.2	40.4	45.8		53.4	51.8	50.0	46.5	
65 to < 75	15.7	25.5	33.9	38.3		26.2	28.8	30.1	36.6	
≥ 75	4.7	11.3	25.7	16.0		20.4	19.4	19.9	16.9	
**Sex (male)**	95.7	92.3	87.9	54.4	1.30	60.6	62.0	60.0	59.6	0.04
**CHA_2_DS_2_-VASc**										
Mean ± SD	2.15 ± 1.52	2.66 ± 1.71	3.81 ± 1.99	3.43 ± 1.78	0.94	3.33 ± 1.88	3.3 ± 1.89	3.34 ± 2	3.4 ± 1.83	0.05
0	9.8	6.0	2.1	2.3		3.6	3.5	4.1	3.0	
1	30.9	23.3	11.0	11.9		15.6	16.2	16.2	12.8	
2	25.3	23.6	16.2	19.6		19.7	19.8	18.7	19.3	
≥ 3	65.9	47.1	70.7	66.2		61.1	60.5	61.0	64.9	
**Comorbidities**										
Hypertension	81.9	83.3	85.9	84.4	0.11	83.2	84.1	84.5	84.9	0.04
Diabetes mellitus	22.9	22.0	24.6	23.5	0.06	20.9	22.8	23.3	23.9	0.07
Dyslipidaemia	38.1	41.7	47.3	48.8	0.22	44.3	44.8	45.1	45.7	0.02
Heart failure	24.5	28.0	36.3	31.1	0.26	34.0	33.0	32.7	33.3	0.02
Prior ischemic stroke	14.1	19.2	29.3	27.0	0.38	25.1	25.1	25.3	25.7	0.01
Prior MI	9.1	10.5	12.5	11.8	0.11	11.4	11.7	11.6	11.9	0.01
PAD	16.5	19.2	23.4	21.4	0.17	21.3	21.6	21.5	21.9	0.01
COPD	14.4	17.3	22.0	19.3	0.20	21.2	20.2	19.9	20.1	0.03
Cancer	2.0	3.8	6.3	8.7	0.30	6.0	6.1	5.7	5.8	0.01
CKD	7.3	11.1	20.2	16.9	0.38	17.2	16.7	16.6	16.9	0.01
**Health examination**										
BMI (kg/m^2^)										
Mean ± SD	24.5 ± 3.4	24.6 ± 3.3	24.4 ± 3.5	24.4 ± 3.3	0.08	24.3 ± 3.5	24.4 ± 3.3	24.4 ± 3.4	24.5 ± 3.1	0.04
≥ 25	42.1	44.4	41.0	40.7		40.7	41.5	41.8	40.8	
**Antithrombotic treatment and other medications**										
Oral anticoagulants	19.2	23.7	28.1	29.6	0.24	26.2	26.9	26.5	27.0	0.01
Warfarin	14.6	16.9	18.8	20.1	0.17	20.3	20.2	20.4	20.5	0.00
NOAC	4.6	6.8	9.3	9.5	0.19	7.8	8.9	8.2	8.5	0.03
Antiplatelet agent	23.8	26.2	26.7	25.0	0.07	24.5	26.0	26.0	26.4	0.04
Aspirin	21.1	22.8	22.5	20.8	0.05	20.2	22.1	22.0	22.1	0.04
P2Y12 inhibitor	5.3	6.7	7.8	7.4	0.10	7.4	7.0	7.4	7.7	0.03
Statin	13.3	15.9	19.2	19.3	0.16	16.5	17.7	17.9	18.1	0.04
**Low income**	18.4	17.5	18.0	16.8	0.04	19.5	18.7	17.9	18.0	0.03

*Categorical variables were presented as a percentage and continuous variables were presented as mean and standard deviation.*

*ASD, absolute standardized difference; BMI, body mass index; CKD, chronic kidney disease; COPD, chronic obstructive pulmonary disease; DBP, diastolic blood pressure; eGFR, estimated glomerular filtration rate; IPTW, inverse probability of treatment weighting; MI, myocardial infarction; NOAC, non-vitamin K antagonist oral anticoagulant; PAD, peripheral artery disease; SBP, systolic blood pressure.*

Patients in the HLS 0 group who had a cluster of three unhealthy lifestyles, including current smoking, current drinking, and lack of regular exercise, were younger, more likely to be men, had lower CHA_2_DS_2_-VASc scores, and had a lower prevalence of comorbidities such as hypertension, diabetes, dyslipidaemia, heart failure, prior ischemic stroke, prior myocardial infarction, peripheral artery disease, chronic obstructive pulmonary disease, cancer, and chronic kidney disease than those in the HLS 3 group who had a cluster of three healthy lifestyles. The HLS 0 group showed a lower proportion of patients with obesity (BMI ≥ 25 kg/m^2^) than the HLS 3 group. The proportion of patients receiving oral anticoagulation treatment was higher in the HLS 1, 2, and 3 groups than in the HLS 0 group.

### Risk of Clinical Outcomes According to Healthy Lifestyle Score

**FIGURE 2:**
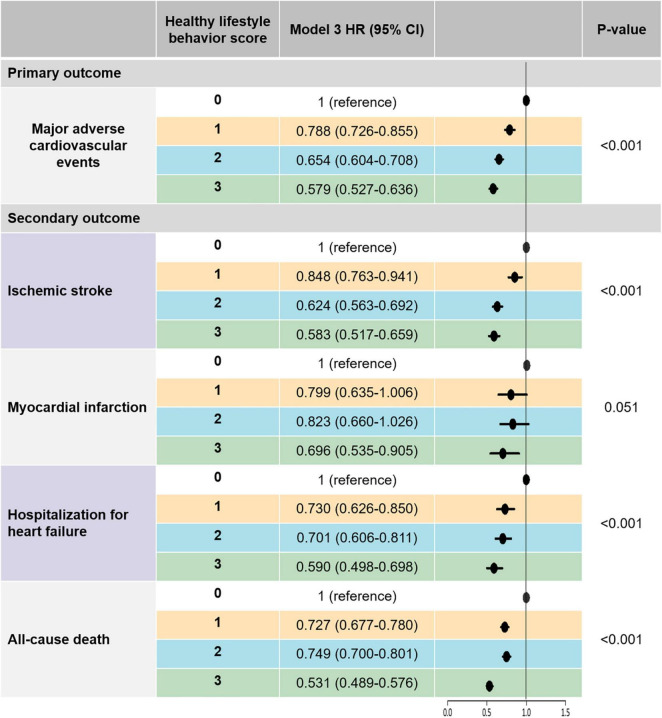
The risk of primary and secondary outcomes according to healthy lifestyle behavior scores: a multivariable-adjusted Cox analysis. Model 3 was adjusted for age, sex, hypertension, diabetes mellitus, dyslipidemia, heart failure, prior ischemic stroke, prior myocardial infarction, peripheral artery disease, chronic obstructive pulmonary disease, cancer, chronic kidney disease, CHA_2_DS_2_-VASc score, use of oral anticoagulant, antiplatelet agent, and statin, BMI, and low income. Major adverse cardiovascular events was defined as the composite outcomes of ischemic stroke, myocardial infarction, and hospitalization for heart failure. BMI, body mass index; CI, confidence interval; HR, hazard ratio.

During a median of 3.5-year follow-up (interquartile range 1.7–5.6), ischemic stroke, myocardial infarction, hospitalization for heart failure, MACE, and all-cause death occurred in 7,110 (3.4%), 1,460 (0.7%), 4,378 (2.1%), 12,298 (16.4%), and 18,318 (8.8%), respectively. Supplementary Table 4 and Figure 2 show the unadjusted and adjusted HRs for clinical outcomes according to HLS groups.

After multivariable adjustment (model 3), patients with HLS 1, 2, and 3 were associated with a lower risk of MACE by 21%, 35%, and 42%, respectively, than those with HLS 0. Consistent results were observed for secondary outcomes. HLS 1, 2, and 3 groups were associated with lower risks of ischemic stroke by 15, 38, and 42%, respectively, compared to the HLS 0 group. The HLS 3 group showed a statistically significant risk reduction for myocardial infarction compared to the HLS 1 group. Compared to the HLS 0 group, the HLS 1, 2, and 3 groups were associated with lower risks of hospitalization for heart failure by 27%, 30%, and 41%, respectively. An increased number of healthy lifestyle behaviors was associated with a lower risk of all-cause death.

### Sensitivity Analysis

**FIGURE 3:**
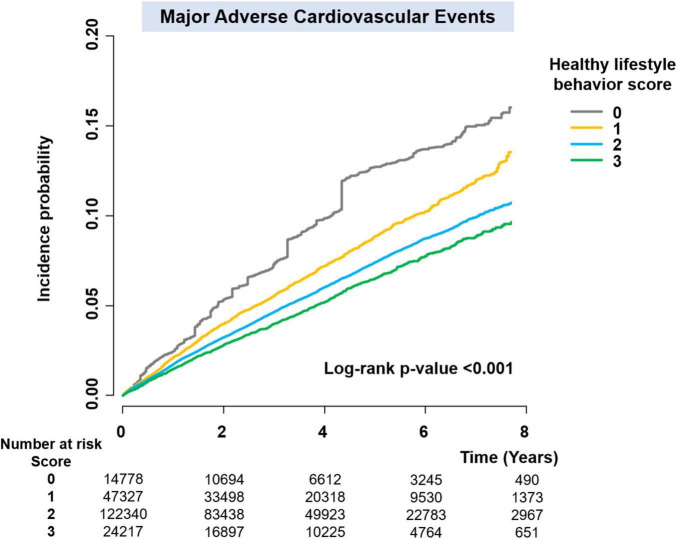
Weighted Kaplan–Meier curves for major adverse cardiovascular events.

**FIGURE 4:**
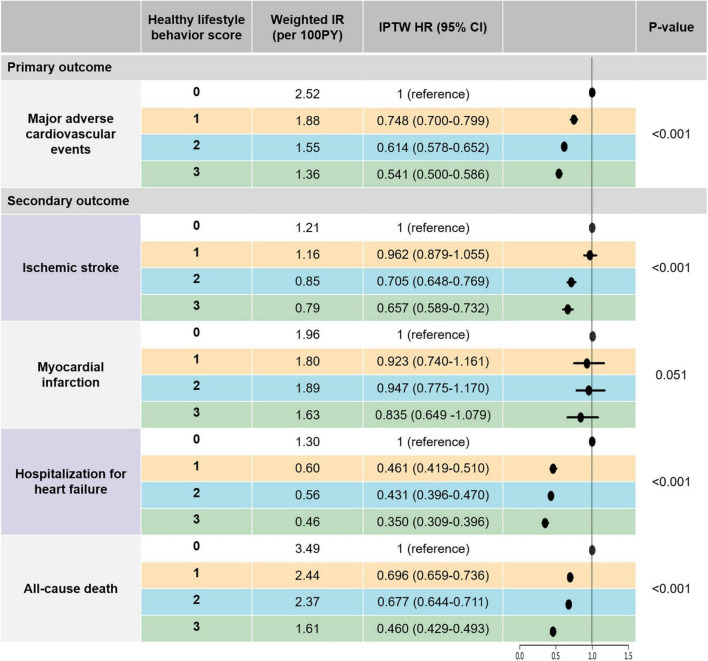
The risk or primary and secondary outcomes according to healthy lifestyle behavior scores: an IPTW analysis. Major adverse cardiovascular events was defined as the composite outcomes of ischemic stroke, myocardial infarction, and hospitalization for heart failure. CI, confidence interval; HR, hazard ratio; IPTW, inverse probability of treatment weighting; IR, incidence rate; PY, person-years.

Since the baseline characteristics of each group stratified by HLS were significantly different, we performed a sensitivity analysis to compare HLS groups for the risk of clinical outcomes using the IPTW method. The baseline covariates were well balanced among the different HLS groups after IPTW (Table 1). Figure 3 and Supplementary Figure 1 reveal weighted Kaplan–Meier curves for clinical outcomes, and Figure 4 shows the weighted incidence rates and HRs for clinical outcomes after IPTW. The results were largely consistent with those of the multivariable Cox analysis (Model 3).

### Subgroup Analyses

The results of subgroup analyses were consistent with the main analysis. For the primary outcome, there was no significant interaction between each subgroup and the risk of MACE according to HLS (Table 2), except for the age subgroups. Although p for interaction was 0.019 in the age subgroup analyses and the HRs were slightly different in different age subgroups, the trends and directionality of HRs of HLS 1, 2, and 3 compared to HLS 0 were consistent in all age subgroups.

**TABLE 2:** Subgroup analyses for major adverse cardiovascular events.

Subgroup	HLS	Number	Events	IR	HR (95% CI)	*p*-value	p-for-interaction
**Age (years)**							
< 65	0	11,760	429	0.96	1 (reference)	< 0.001	0.019
	1	29,903	945	0.83	0.774 (0.690–0.868)		
	2	49,419	1,398	0.72	0.626 (0.558–0.703)		
	3	11,080	259	0.60	0.503 (0.429–0.59)		
65 to < 75	0	2,323	216	2.61	1 (reference)	< 0.0001	
	1	12,071	977	2.29	0.868 (0.749–1.006)		
	2	41,522	2,982	1.98	0.710 (0.616–0.819)		
	3	9,269	575	1.74	0.666 (0.568–0.780)		
≥ 75	0	695	105	4.95	1 (reference)	< 0.001	
	1	5,353	617	3.89	0.765 (0.621–0.941)		
	2	31,399	3,457	3.87	0.673 (0.552–0.820)		
	3	3,868	338	3.03	0.593 (0.475–0.739)		
**Sex**							
Male	0	14,144	720	1.36	1 (reference)	< 0.001	0.380
	1	40,104	2,202	1.51	0.810 (0.744–0.882)		
	2	56,528	3,354	1.72	0.683 (0.628–0.742)		
	3	13,171	684	1.49	0.626 (0.563–0.697)		
Female	0	634	30	1.37	1 (reference)	< 0.001	
	1	7,223	337	1.26	0.657 (0.452–0.954)		
	2	65,812	4,483	1.88	0.521 (0.363–0.747)		
	3	11,046	488	1.17	0.452 (0.313–0.655)		
**CHA_2_DS_2_-VASc score**							
≤ 3	0	12,081	472	1.01	1 (reference)	< 0.001	0.135
	1	33,796	1,242	0.96	0.760 (0.683–0.846)		
	2	57,417	1,874	0.84	0.614 (0.552–0.684)		
	3	13,315	394	0.77	0.541 (0.471–0.621)		
> 3	0	2,697	278	3.16	1 (reference)	< 0.001	
	1	13,531	1,297	2.97	0.825 (0.725–0.940)		
	2	64,923	5,963	2.84	0.699 (0.617–0.791)		
	3	10,902	778	2.15	0.617 (0.537–0.709)		
**Prior ischemic stroke**							
No	0	12,698	540	1.12	1 (reference)	< 0.001	0.691
	1	38,262	1,706	1.20	0.776 (0.704–0.855)		
	2	86,472	4,494	1.41	0.639 (0.581–0.704)		
	3	17,671	720	1.10	0.571 (0.509–0.641)		
Yes	0	2,080	210	2.94	1 (reference)	< 0.001	
	1	9,065	833	2.75	0.796 (0.684–0.927)		
	2	35,868	3,343	2.89	0.669 (0.579–0.774)		
	3	6,546	452	2.03	0.576 (0.487–0.680)		
**OAC**							
No	0	11,940	604	1.31	1 (reference)	< 0.001	0.376
	1	36,122	1,894	1.38	0.764 (0.696–0.838)		
	2	87.923	5,629	1.70	0.643 (0.588–0.703)		
	3	17,058	805	1.23	0.558 (0.501–0.622)		
Yes	0	2,838	146	1.60	1 (reference)	< 0.001	
	1	11,205	645	1.85	0.895 (0.747–1.072)		
	2	3,4417	2,208	2.17	0.720 (0.604–0.857)		
	3	7,159	367	1.66	0.671 (0.552–0.816)		

*Adjusted for age, sex, hypertension, diabetes mellitus, dyslipidemia, heart failure, prior ischemic stroke, prior myocardial infarction, peripheral artery disease, chronic obstructive pulmonary disease, cancer, chronic kidney disease, CHA_2_DS_2_-VASc score, use of oral anticoagulant, antiplatelet agent, and statin, BMI, and low income.*

*CI, confidence interval; HLS, healthy lifestyle behavior score; HR, hazard ratio; IR, incidence rate; OAC, oral anticoagulant.*

## Discussion

In this analysis of a large-scale nationwide population-based cohort study, we sought to determine whether the accumulation of healthy lifestyle behaviors was associated with a lower risk of MACE. Our principal findings were as follows: (1) although the study patients were newly diagnosed with AF, 7% of patients had a cluster of three unhealthy lifestyles, including current smoking, current drinking, and lack of regular exercise, whereas 12% of patients had all three healthy lifestyle behaviors; (2) healthy lifestyle behaviors as a composite score were associated with a substantially lower risk of MACE, including ischemic stroke, myocardial infarction, and hospitalization for heart failure, and all-cause death; and (3) there was an inverse dose-response relationship in which an increased number of healthy lifestyle behaviors were associated with lower risks of MACE.

Considering that healthy lifestyle factors are not isolated but often occur in a cluster with other healthy lifestyle factors, we suggest that an integrated approach to lifestyle modification is needed to improve the clinical outcomes in patients with AF.

Traditionally, the management of patients with AF has been particularly focused on the prevention of stroke with anticoagulation and control rate and rhythm for symptom relief. A more integrated care approach that addresses modifiable risk factors, including coexisting cardiovascular comorbidities and unhealthy lifestyle behaviors, has been promoted in recent guidelines (7, 29). A recent scientific statement from the American Heart Association comprehensively summarizes the modifiable risk factors for primary and secondary prevention of AF, similar to the statement from the European Heart Rhythm Association (29, 30). Moderate exercise, smoking cessation, and alcohol intake reduction are recommended to reduce the risk of AF occurrence and reduce the AF burden (29).

Several previous observational studies have established that healthy lifestyle behaviors are associated with reducing the AF burden and lower risks of adverse clinical outcomes (11, 12, 14, 15, 31, 32). Subsequently, well-designed randomized controlled trials (RCTs) have strengthened previous findings in observational studies (13, 33). Although an RCT could not be conducted for smoking, several observational studies have shown that smoking was associated with an increased risk of stroke and death, and smoking cessation after AF diagnosis might reduce the risk of stroke and MACE (11, 12, 32). Alcohol is a well-known risk factor for the development of AF (34) and is associated with an increased risk of thromboembolic events, including ischemic stroke and AF hospitalization (14). Indeed, a recent RCT further confirmed the impact of abstinence from alcohol on reducing AF recurrence and its burden (13). In patients with AF, performing regular exercise is associated with a lower risk of all-cause death (15). In addition, higher physical activity and cardiorespiratory fitness have been associated with a lower long-term risk of death from cardiovascular disease (a composite of myocardial infarction, heart failure, and stroke) and all-cause death (31). Of note, cardiorespiratory fitness was also closely related to AF recurrence risk, with a significant dose-response relationship (33).

It must be emphasized that much of the evidence in previous studies has focused on an isolated component of lifestyle behaviors (11–15, 31–33). However, healthy (or unhealthy) lifestyle behaviors tend to cluster (16–19, 35, 36). Two RCTs have shown that intensive implementation of integrated care for modifiable risk factors significantly reduced AF recurrence (37, 38). Aggressive risk factor management, including blood pressure control, weight management, lipid management, glycemic control, sleep-disordered breathing management, smoking cessation, and alcohol reduction by the physician-directed clinic after AF catheter ablation reduced AF recurrence and symptom severity with left atrial reverse remodeling (37). In patients with early persistent AF and mild-to-moderate heart failure, optimal medical therapy combined with cardiac rehabilitation, including physical activity, dietary restriction, and counseling, improves sinus rhythm maintenance (38). In these studies, the study population was perhaps more selected from the general AF population. The components of lifestyle intervention were slightly different from our study, and the primary outcome was AF recurrence, not a hard adverse clinical outcome.

According to our results (Supplementary Table 1), current smoking is the most powerful factor to be associated with a higher risk of MACE [adjusted HR 1.479, 95% confidence interval (CI) 1.393–1.571]. Followed by current smoking, current drinking was associated with a higher risk of MACE by 16–32% [adjusted HR 1.159 (95% CI 1.082–1.241), 1.261 (1.139–1.397), and 1.317 (1.183–1.466) for mild, moderate, and heavy drinker, respectively]. In the case of regular physical activity, although it depends on the degree (or amount) of exercise, lack of exercise was associated with a higher risk of MACE by 10–20%. In this analysis, we did not weight each lifestyle behavior due to primarily exploring the impact of the combination of unhealthy lifestyle behavior on the risk of MACE. Further study is needed to weight each lifestyle behavior by considering the dose-response relationship of each factor and develop a more sophisticated lifestyle score for predicting AF-related adverse.

In subgroup analyses, among relatively low-risk patients with CHA_2_DS_2_-VASc ≤ 3, a cluster of healthy lifestyle behaviors was associated with a significantly lower risk of MACE. In addition, the benefit of a healthy lifestyle cluster was consistently observed regardless of whether the patients were anticoagulated or not. According to a recent study, current smoking was a predictor for future ischemic stroke in low-risk patients who were not indicated oral anticoagulation (male with CHA_2_DS_2_-VASc 0 or female with CHA_2_DS_2_-VASc 1) (39). Hence, proactive lifestyle risk evaluation and promotion of a healthy lifestyle in the early period of AF diagnosis would also be beneficial to prevent stroke in low-risk patients who are not indicated for oral anticoagulation treatment immediately.

The European Hypertension Guidelines regarded AF as an equivalent of cardiovascular disease risk (40). Hence, the comprehensive management of AF patients should include not only oral anticoagulation therapy but also general control or cardiovascular risk factors and comorbidities (7). The time point of immediate after the new diagnosis of AF could be the best chance to promote healthy lifestyle behaviors, including smoking cessation, reducing alcohol consumption, and initiating regular exercise, to improve clinical outcomes and reduce AF-related adverse events in the future. Physician-directed proactive management of modifiable lifestyle risk factors should be more emphasized in a holistic approach to AF care, as recommended in the guidelines (7). Indeed, lifestyle optimization is the C of the ABC (Atrial fibrillation Better Care) pathway whereby ABC pathway compliant care has been shown in numerous studies to be associated with better clinical outcomes (15, 41–43).

### Study Limitations

This study included a large number of patients with incident AF. It comprehensively analyzed the association between an increased number of healthy lifestyle behaviors and the risk of MACE with sufficient statistical power. However, there are several limitations to this study. First, we classified healthy lifestyle behaviors using self-reported questionnaires. Although several studies have reported the association between lifestyle factors and adverse clinical outcomes using self-reported questionnaires, recall bias could be one of the major potential limitations (19, 22, 23, 31, 33). Second, although we performed multivariable adjustment and balanced the baseline characteristics of the different groups using IPTW, we cannot exclude the possibility of unmeasured confounding factors. For example, the types of AF, such as the paroxysmal and persistent, or the actual burden of AF at baseline could not be measured in this database. Third, changes in baseline variables during follow-up, including lifestyle factors and medication use, were not considered in this analysis. Lastly, in this study, covariates and study outcomes were defined based on the diagnostic codes, which could be affected by the physicians’ clinical practice, thus, might have resulted in an underestimation and overestimation. To overcome this limitation, we used a well-established and widely used operational definition in previous studies, or a validated definition through our own data (21, 44).

## Conclusion

Increased number of healthy lifestyle behaviors, including quitting smoking, abstaining from alcohol consumption, and performing regular physical activity, were significantly associated with lower risks of MACE and all-cause death in patients with new-onset AF. These findings support the promotion of a healthy lifestyle and a more holistic or integrated approach to reduce the risk of adverse events in patients with AF.

## Data Availability Statement

Publicly available datasets were analyzed in this study. This data can be found here: http://nhiss.nhis.or.kr/bd/ab/bdaba000eng.do.

## Ethics Statement

The studies involving human participants were reviewed and approved by Seoul National University Hospital Institutional Review Board. Written informed consent for participation was not required for this study in accordance with the national legislation and the institutional requirements.

## Author Contributions

S-RL, E-KC, and GL: conceptualization. S-WL and K-DH: methodology and data curation. K-DH: software and resources. S-RL, S-WL, S-HP, and E-KC: validation. S-WL: formal analysis. S-RL: investigation, writing—original draft preparation, and visualization. S-RL, S-HP, E-KC, and GL: writing—review and editing. SO: supervision. E-KC: project administration and funding acquisition. All authors have read and agreed to the published version of the manuscript.

## Conflict of Interest

E-KC: research grants or speaking fees from Bayer, BMS/Pfizer, Biosense Webster, Chong Kun Dang, Daiichi-Sankyo, Dreamtech Co., Ltd., Medtronic, Samjinpharm, Sanofi-Aventis, Seers Technology, Skylabs, and Yuhan. GL: consultant and speaker for BMS/Pfizer, Boehringer Ingelheim and Daiichi-Sankyo, but no fees are received personally; no other relationships or activities that could appear to have influenced the submitted work. The remaining authors declare that the research was conducted in the absence of any commercial or financial relationships that could be construed as a potential conflict of interest.

## Publisher’s Note

All claims expressed in this article are solely those of the authors and do not necessarily represent those of their affiliated organizations, or those of the publisher, the editors and the reviewers. Any product that may be evaluated in this article, or claim that may be made by its manufacturer, is not guaranteed or endorsed by the publisher.

## Abbreviations

AF: atrial fibrillation
ASD: absolute standardized difference
CI: confidence interval
HLS: healthy lifestyle behavior score
HR: hazard ratio
IPTW: inverse probability of treatment weighting
MACE: major adverse cardiovascular event
NHIS: National Health Insurance Service.
