# Supplementary material for: Clustering of Unhealthy Lifestyle and the Risk of Adverse Events in Patients With Atrial Fibrillation
Source: Front Cardiovasc Med. 2022 Jul 4;9:885016. doi: 10.3389/fcvm.2022.885016 (PMC9289142; doi:10.3389/fcvm.2022.885016)
Supplement: Supplementary file 1 [file Data_Sheet_1.pdf]

frontiers

Supplementary Material

Supplemental Tables

Supplementary Table 1. Association between each unhealthy lifestyle behavior and the risk of major adverse cardiovascular events

	Number	Event	IR	Adjusted HR (95% CI)	p-value
**Smoking**					
**Non-smoker**	126,467	7895	1.73	1 (reference)	<0.001
**Ex-smoker total**	52,756	2668	1.44	0.971 (0.921-1.022)	
**Ex-smoker, >0 to <10 PY**	13,831	578	1.17	0.914 (0.837-0.999)	
**Ex-smoker, >10 to <20 PY**	13,102	583	1.26	0.939 (0.859-1.026)	
**Ex-smoker, >20 to <30 PY**	9808	494	1.44	0.993 (0.902-1.092)	
**Ex-smoker ≥30 PY**	16,015	1013	1.83	1.017 (0.947-1.092)	
**Current smoker total**	29,439	1735	1.60	1.479 (1.393-1.571)	
**Current smoker >0 to <10 PY**	6370	312	1.30	1.457 (1.297-1.636)	
**Current smoker >10 to <20 PY**	7703	408	1.47	1.503 (1.355-1.668)	
**Current smoker >20 to <30 PY**	6221	387	1.69	1.531 (1.376-1.704)	
**Current smoker ≥30 PY**	9145	628	1.87	1.446 (1.325-1.578)	
**Alcohol consumption**					
**Non-drinker**	142,753	9148	1.81	1 (reference)	<0.001
**Mild drinker (>0 to <105 per week)**	38,773	1820	1.28	1.159 (1.082-1.241)	
**Moderate drinker (>105 to <210 per week)**	14,955	691	1.24	1.261 (1.139-1.397)	
**Heavy drinker (≥210 per week)**	12,181	639	1.41	1.317 (1.183-1.466)	
**Exercise**					
**Non-exerciser**	155,126	9774	1.77	1 (reference)	<0.001
**MPA, 3-7 days /week**	19,422	953	1.37	0.905 (0.846-0.968)	
**VPA, 3-7 days /week**	34,114	1571	1.25	0.889 (0.842-0.938)	
**Non-exerciser**	183,560	11,025	1.68	1 (reference)	<0.001
**MPA, 5-7 days/week**	9882	498	1.41	0.881(0.805-0.964)	
**VPA, 5-7 days/week**	15,220	775	1.39	0.904(0.840-0.973)	
**Non-exerciser**	115,196	7921	1.95	1 (reference)	<0.001
**MVPA 1-2 days/week**	39,930	1853	1.26	0.959 (0.911-1.010)	

**MVPA 3-4 days/week**	28,434	1251	1.21	0.899 (0.846-0.955)	
**MVPA 5-6 days/week**	15,044	709	1.30	0.893 (0.826-0.965)	
**MVPA ≥7 days/week**	10,058	564	1.54	0.854 (0.783-0.93)	
**Non-exerciser**	57,595	4414	2.16	1 (reference)	<0.001
**0 to <500 MET-min /week**	57,159	3152	1.52	0.897 (0.856-0.939)	
**500 – 999 MET-min/week**	57,063	3027	1.48	0.872 (0.832-0.914)	
**1000 – 1499 MET-min/week**	23,052	1043	1.25	0.825 (0.770-0.884)	
**≥1500 MET-min/week**	13,793	662	1.33	0.808 (0.744-0.878)	

IR, per 100 person-years.

Adjusted age, sex, hypertension, diabetes, dyslipidemia, heart failure, prior ischemic stroke, prior myocardial infarction, peripheral artery disease, chronic obstructive pulmonary disease, cancer, chronic kidney disease, CHA_2DS_2-VASc score, oral anticoagulants, antiplatelets, statin, body mass index, and low income. When estimating adjusted HR for smoking, current drinking and regular exercise were additionally adjusted. Similarly, when estimating adjusted HR for drinking, current smoking and regular exercise were additionally adjusted. When estimating adjusted HR for exercise, current smoking and current drinking were additionally adjusted.

Abbreviation: CI, confidence interval; HR, hazard ratio; IR, incidence rate; MET, metabolic equivalent of task; MPA, moderate intensity of physical activity; MVPA, moderate or vigorous intensity of physical activity; PY, pack-year; VPA, vigorous intensity of physical activity.

Supplementary Table 2. Definition of covariates and outcomes

Diagnosis	ICD-10-CM code and definition	Diagnostic definition
**Inclusion/exclusion criteria**		
**Atrial fibrillation**	I48.0-48.4, I48.9	Admission or outpatient department≥1
**Valvular atrial fibrillation**	I05.0, I05.2, I05.9, Z95.2-Z95.4	Admission or outpatient department≥1
**Comorbidities**		
**Hypertension**	I10-I13, I15; and minimum 1 prescription of anti-hypertensive drug (thiazide, loop diuretics, aldosterone antagonist, alpha-/beta-blocker, calcium-channel blocker, angiotensin-converting enzyme inhibitor, angiotensin II receptor blocker).	Admission≥1 or outpatient department≥1
	Or systolic/diastolic blood pressure ≥ 140/90 mmHg	Index health examination
**Diabetes mellitus**	E11-E14; and minimum 1 prescription of anti-diabetic drugs (sulfonylureas, metformin, meglitinides, thiazolidinediones, dipeptidyl peptidase-4 inhibitors, α-glucosidase inhibitors, and insulin).	Admission≥1 or outpatient department≥1
	Or fasting glucose level ≥ 126 mg/dL	Index health examination
**Dyslipidemia**	E78	Admission or outpatient department≥1
	Or Total cholesterol ≥ 240 mg/dL	Index health examination
**Heart failure**	I50	Admission or outpatient department≥1
**Prior stroke**	I63, I64	Admission or outpatient department≥1
**Vascular disease**		
**Prior myocardial infarction**	I21, I22	Admission or outpatient department≥1
**Peripheral artery disease**	I70, I73	Admission or outpatient department≥1
**Chronic kidney disease**	Estimated glomerular filtration rate <60 ml/min/1.73m^2	Index health examination
**Chronic obstructive pulmonary disease**	J41-44	Admission or outpatient department≥1
**Cancer**	C00-97 and RID code (V193)	Admission or outpatient department≥1
**Scores**		
**CHA_2DS_2-VASc score**	Heart failure (1 point), hypertension (1 point), age ≥75 years (2 points), diabetes (1 point), previous stroke/systemic embolism/transient ischemic attack (2 points), vascular disease (prior MI or PAD, 1 point) and female sex (1 point)	
**Clinical outcome**		

**Ischemic stroke**	I63, I64	Primary diagnosis, admission≥1 (≥3 days) and brain imaging (CT or MRI) ≥1
**Myocardial infarction**	I21, I22	Primary diagnosis, admission≥1
**Hospitalization for heart failure**	I50	Primary diagnosis, admission≥1
**Major adverse cardiovascular events**	Ischemic stroke + myocardial infarction + hospitalization for heart failure	Each definition was described as above.

Abbreviation: ICD, international classification of disease; CM, clinical modification.

Supplementary Table 3. Baseline characteristics of the total study population

	**Total (N=208,662)**
**Unhealthy lifestyle behavior**	
**Current smoker**	29,439 (14.1)
**Any drinker**	65,909 (31.6)
**Lack of regular exercise**	165,990 (79.5)
**Healthy lifestyle behavior score**	
**0**	14,778 (7.1)
**1**	47,327 (22.7)
**2**	122,340 (58.6)
**3**	24,217 (11.6)
**Age, years**	
**Mean ± SD**	63.7±12.7
**<65**	102,162 (49.0)
**65 to <75**	65,185 (31.2)
**≥75**	41,315 (19.8)
**Sex (male)**	123,947 (59.4)
**CHA_2DS_2-VASc**	
**Mean ± SD**	3.38±1.96
**0**	7,412 (3.6)
**1**	31,859 (15.3)
**2**	39,489 (18.9)
**≥3**	129,902 (62.3)
**Comorbidities**	
**Hypertension**	177,067 (84.9)
**Diabetes mellitus**	49,518 (23.7)
**Dyslipidemia**	95,059 (45.6)
**Heart failure**	68,742 (32.9)
**Prior ischemic stroke**	53,559 (25.7)
**Prior myocardial infarction**	24,432 (11.7)
**Peripheral artery disease**	45,353 (21.7)
**Chronic obstructive pulmonary disease**	41,937 (20.1)
**Cancer**	11,956 (5.7)
**Chronic kidney disease**	35,131 (16.8)

**Health examination**	
**Body mass index (kg/m²)**	
**Mean ± SD**	24.4±3.4
**≥25**	87,252 (41.8)
**Fasting glucose (mg/dL)**	105.3±29.0
**Systolic blood pressure (mmHg)**	125.9±15.8
**Diastolic blood pressure (mmHg)**	77.1±10.4
**Estimated GFR (mL/min)**	79.8±30.4
**Antithrombotic treatment**	
**Oral anticoagulants**	55,619 (26.7)
**Warfarin**	38,081 (18.3)
**NOAC**	17,538 (8.4)
**Antiplatelet agent**	54,703 (26.2)
**Aspirin**	46,403 (22.2)
**P2Y12 inhibitor**	15,265(7.3)
**Statin**	37,614(18.0)
**Low income**	37,153 (17.8)

Abbreviation: GFR, glomerular filtration rate; NOAC, non-vitamin K antagonist oral anticoagulant.

Supplementary Table 4. Hazard ratios for primary and secondary outcomes according to the combination of healthy lifestyle behavior

Healthy lifestyle behavior score	Number	Event	IR	Model 1<br>HR (95% CI)	Model 2<br>HR (95% CI)	Model 3<br>HR (95% CI)
**Primary outcome**						
**Major adverse cardiovascular events**						
**0**	14,778	750	1.36	1 (reference)	1 (reference)	1 (reference)
**1**	47,327	2,539	1.47	1.083 (0.998-1.175)	0.777 (0.716-0.844)	0.788 (0.726-0.855)
**2**	122,340	7,837	1.81	1.328 (1.232-1.431)	0.667 (0.616-0.722)	0.654 (0.604-0.708)
**3**	24,217	1,172	1.34	0.984 (0.897-1.078)	0.566 (0.516-0.622)	0.579 (0.527-0.636)
**p-value**				<0.001	<0.001	<0.001
**Secondary outcomes**						
**Ischemic stroke**						
**0**	14,778	455	0.81	1 (reference)	1 (reference)	1 (reference)
**1**	47,327	1,623	0.93	1.142 (1.029-1.267)	0.836 (0.753-0.928)	0.848 (0.763-0.941)
**2**	122,340	4,338	0.98	1.208 (1.097-1.330)	0.630 (0.568-0.698)	0.624 (0.563-0.692)
**3**	24,217	694	0.78	0.961 (0.854-1.082)	0.570 (0.505-0.643)	0.583 (0.517-0.659)
**p-value**				<0.001	<0.001	<0.001
**Myocardial infarction**						
**0**	14,778	98	1.73	1 (reference)	1 (reference)	1 (reference)
**1**	47,327	302	1.70	0.985 (0.784-1.237)	0.791 (0.629-0.995)	0.799 (0.635-1.006)
**2**	122,340	915	2.04	1.181 (0.959-1.455)	0.853 (0.685-1.063)	0.823 (0.660-1.026)
**3**	24,217	145	1.61	0.934 (0.723-1.207)	0.710 (0.547-0.922)	0.696 (0.535-0.905)
**p-value**				0.004	0.040	0.051
**Hospitalization for heart failure**						
**0**	14,778	217	0.38	1 (reference)	1 (reference)	1 (reference)
**1**	47,327	738	0.42	1.087 (0.935-1.265)	0.724 (0.622-0.843)	0.730 (0.626-0.850)
**2**	122,340	3,028	0.68	1.771 (1.543-2.032)	0.729 (0.631-0.843)	0.701 (0.606-0.811)
**3**	24,217	395	0.44	1.148 (0.973-1.354)	0.574 (0.485-0.680)	0.590 (0.498-0.698)
**p-value**				<0.001	<0.001	<0.001
**All-cause death**						

**0**	14,778	1,004	1.76	1 (reference)	1 (reference)	1 (reference)
**1**	47,327	3,320	1.87	1.061 (0.988-1.138)	0.692 (0.645-0.743)	0.727 (0.677-0.780)
**2**	122,340	12,533	2.79	1.588 (1.498-1.693)	0.769 (0.719-0.822)	0.749 (0.700-0.801)
**3**	24,217	1,461	1.62	0.923 (0.852-1.001)	0.524 (0.483-0.569)	0.531 (0.489-0.576)
**p-value**				<0.001	<0.001	<0.001

IR, per 100 person-years.

Abbreviation: CI, confidence interval; HR, hazard ratio; IR, incidence rate.
