# Supplementary figures and images for: Clustering of Unhealthy Lifestyle and the Risk of Adverse Events in Patients With Atrial Fibrillation
Source: Front Cardiovasc Med. 2022 Jul 4;9:885016. doi: 10.3389/fcvm.2022.885016 (PMC9289142; doi:10.3389/fcvm.2022.885016)

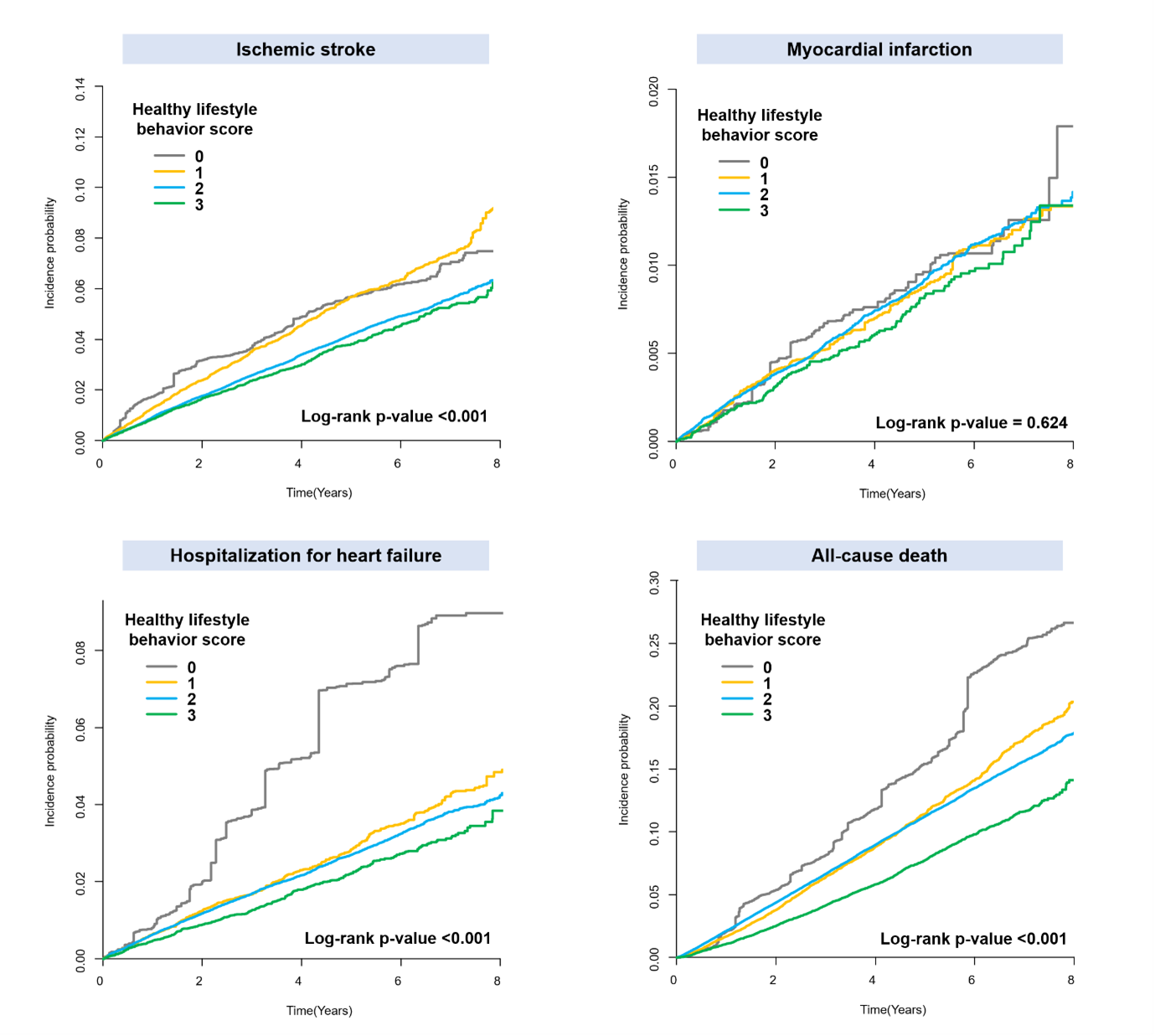

Supplement: Supplementary file 2 [file Image_1.TIF]
